# Breast Mammographic Density: Stromal Implications on Breast Cancer Detection and Therapy

**DOI:** 10.3390/jcm9030776

**Published:** 2020-03-12

**Authors:** Patricia Fernández-Nogueira, Mario Mancino, Gemma Fuster, Paloma Bragado, Miquel Prats de Puig, Pere Gascón, Francisco Javier Casado, Neus Carbó

**Affiliations:** 1 Institut d’Investigacions Biomèdiques Augustí Pi i Sunyer (IDIBAPS), 08036 Barcelona, Spain; 2 Department of Biochemistry and Molecular Biomedicine, University of Barcelona, Institute of Biomedicine, University of Barcelona (IBUB), 08028 Barcelona, Spain; 3 Department of Medicine, University of Barcelona, 08036 Barcelona, Spain; 4 Department of Biochemistry & Physiology, School of Pharmacy and Food Sciences, University of Barcelona, 08028 Barcelona, Spain; 5 Department of Biosciences, Faculty of Sciences and Technology, University of Vic, 08500 Vic, Spain; 6 Department of Biochemistry and Molecular Biology, Faculty of Pharmacy, Complutense University of Madrid, Health Research Institute of the Hospital Clínico San Carlos, 28040 Madrid, Spain; 7 Breast Committee, Hospital El Pilar, Quirón salud Group, 08006 Barcelona, Spain; 8 Oncology and Multidisciplinary Knowledge, 08036 Barcelona, Spain

**Keywords:** tumour stroma, mammographic density, therapy resistance, ductal carcinoma in situ (DCIS), invasive ductal carcinoma (IDC), breast cancer detection

## Abstract

Current evidences state clear that both normal development of breast tissue as well as its malignant progression need many-sided local and systemic communications between epithelial cells and stromal components. During development, the stroma, through remarkably regulated contextual signals, affects the fate of the different mammary cells regarding their specification and differentiation. Likewise, the stroma can generate tumour environments that facilitate the neoplastic growth of the breast carcinoma. Mammographic density has been described as a risk factor in the development of breast cancer and is ascribed to modifications in the composition of breast tissue, including both stromal and glandular compartments. Thus, stroma composition can dramatically affect the progression of breast cancer but also its early detection since it is mainly responsible for the differences in mammographic density among individuals. This review highlights both the pathological and biological evidences for a pivotal role of the breast stroma in mammographic density, with particular emphasis on dense and malignant stromas, their clinical meaning and potential therapeutic implications for breast cancer patients.

## 1. Introduction

Breast cancer (BC), impacting over 2 million women each year, is the most common cancer occurring in women and constitutes the second most frequent cancer overall. In 2018, 627000 women died because of BC [1], the greatest number of cancer-related deaths in women. In this scenario, growing evidence suggests that the percentage of mammographic density (MD), a concept first described in the 1970s and obtained by weighing the proportion of high dense (stromal, epithelial) and low dense (adipose) tissue, can be a risk factor for BC. MD has been positively associated with tumour size, lymph node status and lymphatic or vascular invasion [2], and it may hamper tumour detection. The mammary gland constitutes a complex structure in which mammary epithelial cells are embedded in a stroma composed of different types of cells (including adipose cells, immune cells, fibroblasts, lymphatic and blood vessels) and an intricate extracellular matrix (ECM). This stroma regulates the proliferation, differentiation and survival of the epithelial cells through a complex network of interactions [3]. The microenvironment of the normal mammary tissue can also act as a barrier to tumour growth and exert restraining forces that prevent tumorigenesis [4].

The relative abundance within the breast of low-density adipose tissue vs. high-density glandular and fibroblastic stromal tissue and ECM determines the MD of every single individual [5,6]. The composition of the mammary gland experiences dramatic changes along the life of women (expansion and development during puberty, repetitive proliferation and apoptosis episodes during menstrual cycle, full development of alveoli during lactation). Because of this dynamic and flexible scenario, a significant feature of MD compared to other well-known risk factors is that it is modifiable and, as a consequence of this plasticity, the reduction of breast density would be a valuable strategy to prevent cancer onset.

Homeostasis in this kind of dynamic tissues imposes a strict control between cell proliferation and cell death. The maintenance of this balance depends critically upon the intercellular communication, not only between ductal epithelial cells and stroma cells, but also with elements of another important regulator of tissue homeostasis and normal cell behaviour, the ECM. A correct stable tissue architecture must rely upon tight junction and cell adhesion molecules that anchor cells to the ECM, such as β1 integrins or E-cadherin. Ensuring a correct organ homeostasis can help preventing neoplastic transformation [7].

## 2. Breast Cancer and Mammographic Density

**Figure 1:**
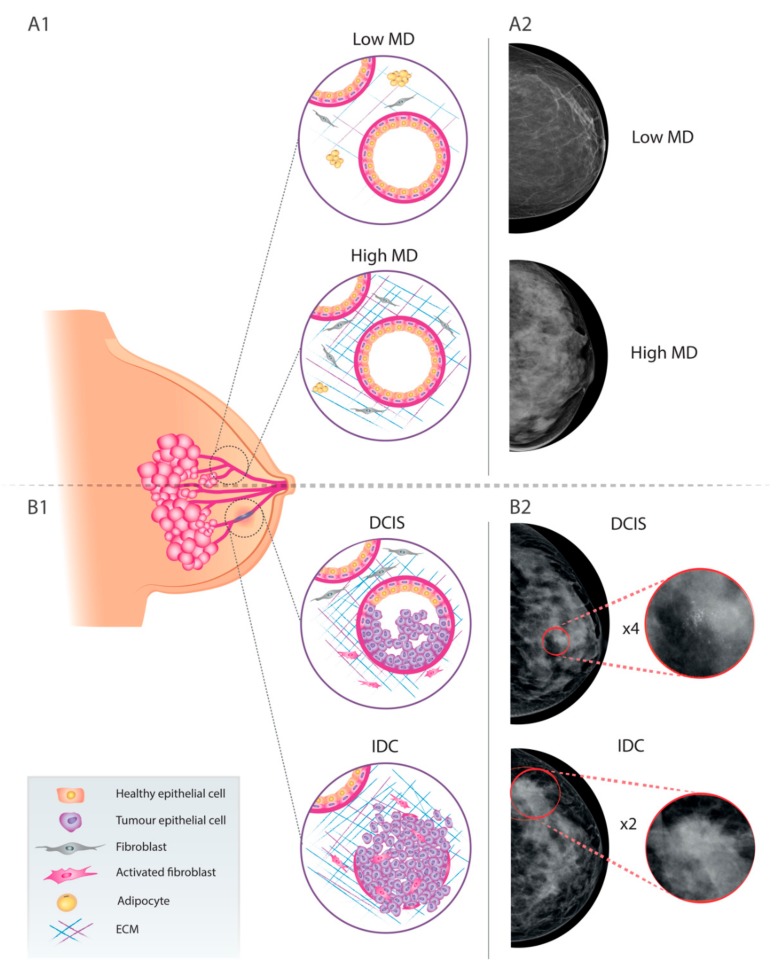
Schematic representation of mammary gland organisation and representative mammograms of healthy breast tissue, including low (**A**1) and high (**A**2) mammographic density areas; and malignant lesions, including DCIS (**B**1) and IDC (**B**2). MD: mammographic density; DCIS: ductal carcinoma in situ; IDC: invasive ductal carcinoma.

The specific MD of every single woman has been shown to be a major independent risk factor for breast cancer. Even though the reported results show an outstanding variability, high breast cancer density has been correlated with larger tumours and with positive lymph nodes [8]. However, the sensitivity of a mammogram is subjected to the density of the breast tissue [9]. In general, women with high breast density (75% or more of MD due to a higher number of stromal and epithelial cells and less fatty adipose tissue) have a 4–6 fold increased risk to develop BC in their lifetime compared with those with low breast density (10% or less of MD due to a higher amount of fatty adipose tissue). The different components of breast tissue react in a different way to X-rays. Fat tissue is relatively translucent, since it absorbs few X-rays and thus it results in dark areas on the image. On their hand, epithelial and stromal tissues filter X-rays more efficiently, absorbing their energy and thus appearing as clear areas (Figure 1A1,A2).

Breast lesions are not easily discernible in these areas since dense tissue and tumours both appear as white areas on a screening mammogram (Figure 1B1,B2). So, the lack of contrast between cancer and healthy tissue may jeopardise the detection of BC in case of high MD, generating false positives and false negatives [5].

Therefore, outcomes obtained through screening mammography of highly dense breast tissue seem to be less effective and/or inconclusive in discovering suspicious lesions and probably lead to late-stage diagnosis [8]. Nonetheless, in spite of this drawback, mammography continues to be the most commonly method used for BC detection, but even the newest improvements to this technique, such as full field digital mammography (FFDM) or digital breast thomosynthesis (DBT), cannot completely overcome the occurrence of false negatives in women with high MD [10]. Moreover, to increase breast cancer detection and benefit women with exceedingly dense breasts, a supplemental tailored breast screening strategy, such as magnetic resonance imaging (MRI), in between conventional screening mammograms is a valuable option. Recent findings demonstrate that MRI screenings is capable to minimize false positive outcomes compared to normal mammography alone [11]. Unfortunately the high cost of this technique is threatening the implementation of MRI screening as a routine control strategy. To this respect, different approaches/protocols, such as ultrafast, 3-min breast MRI, are under evaluation to reduce cost and improve access and tolerance as well [12].

A large number of studies show a strong positive correlation between a high dense breast and the risk of developing BC beyond the mere possibility of interfering with screening mammography results [13,14,15,16]. Not only detection of tumours is more difficult in women with dense breast tissue, but also tumours might grow quickly between examinations [17]. A critical factor seems to be the MD status when BC is diagnosed. A low MD is usually reported to be associated with a better BC outcome, with a lower risk of local recurrence, although it does not seem to affect neither the risk of metastasis nor the mortality specifically associated to BC [17].

**Figure 2:**
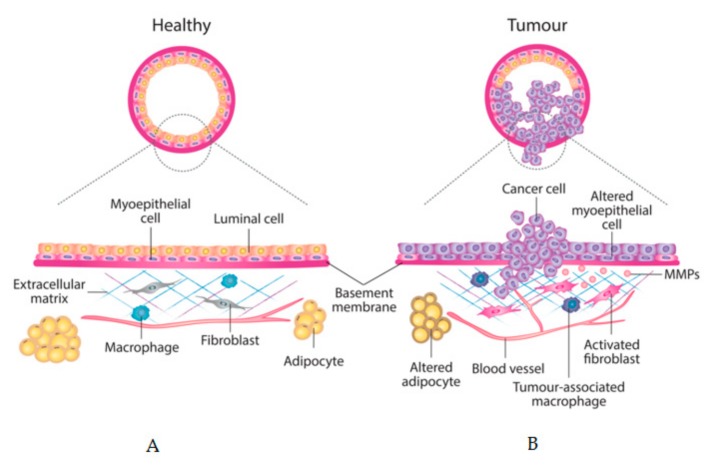
Schematic model of breast epithelium and its stroma. Major changes in cell types and in extracellular matrix (ECM) between: (**A**) healthy mammary gland and (**B**) invasive breast carcinoma are depicted.

However, so far the biological mechanisms underlying the phenomenon of how breast density increases the risk of breast cancer have proven elusive and it is upon this point that recent studies of the interactions between cancer cells and stroma can shed some new light. When the normal network of cell-cell signalling is disrupted, the changes in microenvironment can create a permissive milieu for tumour growth. New players such as mutagens, inflammatory molecules, cytokines and growth factors and other promoting forces, acting alone or in combination, can break the myoepithelial and basement membrane barrier prompting tumour formation [4] (Figure 2). Thus, in this new pro-inflammatory environment, stromal fibroblasts can upregulate in a continuous manner matrix metalloproteinases (MMPs) and other enzymes that can provoke a disruption of the ECM. This breach can be followed by an invasion of immune cells that can in turn overproduce different factors, which will end up promoting abnormal proliferation and invasion [7]. Once initiated, this process will progress until the normal organisation of the tissue collapses and functional disorders may appear. In this new context, any pre-existing epithelial cells with tumorigenic potential can find favourable conditions to proliferate [18]. Cancer cells can now proliferate and interact with this new microenvironment, thus promoting or enhancing abnormal interactions. When this point is reached, the tumour can be considered as a different organ from the original one, embedded in a new permissive environment that it can help create and maintain.

Paradoxically, as the age progresses, MD shows a tendency to decrease while breast cancer incidence generally increases. This apparent contradiction may be explained considering the model of breast cancer incidence proposed by Pike, which contemplates that the real breast tissue age is the result of its cumulative lifetime exposure to hormones and growth factors rather than its chronological age. So, for describing breast cancer’s incidence, this real breast tissue age should be the appropriate measure to apply [19]. It is especially evident in breast cancers associated to persistent hormones exposure (such as oestrogen in advanced age first-time mothers) which usually have a higher degree of MD [20]. Another significant positive link between hormones, high MD and breast cancer incidence is represented by the follicular-phase oestradiol level which is associated with invasive and ER+/PR+ breast cancer in premenopausal women [21].

## 3. Hormonal Therapy and Mammographic Density

Regarding the specific implication of MD in breast cancer therapy, most of the research has been done in the field of hormone treatments. Oestrogenic activity strongly influences MD, and accordingly MD can change in response to tamoxifen anti-oestrogen treatment. However, the results obtained using other oestrogen receptor modulators or aromatase inhibitors have proven less conclusive [22]. A relation between adjuvant therapy and MD changes among women with BC have been described [23]. Patients under adjuvant tamoxifen experienced a higher MD decline than patients who did not received hormonal therapy [23]. Other studies have shown that this decline in MD due to tamoxifen treatment results in a reduced BC risk when administered as chemopreventive treatment and in better BC outcomes (such as a lower risk of recurrence and lower rates of BC specific death) when in adjuvant settings [23]. Other studies have demonstrated that tamoxifen-associated MD decline translates into reduced breast cancer risk in the chemopreventive setting and improved breast cancer outcomes, including reduced risk of recurrence and breast cancer specific death, in the adjuvant settings [22,24]. Once BC is diagnosed, a diminution of MD has also been proposed as a prognostic marker for better long-term survival among patients that have received adjuvant therapy [25].

Nevertheless, discrepant literature can be also found in terms of MD decline as a response of tamoxifen treatment. This is mainly due to poor adherence of patients to treatment, as a consequence of adverse side effects, which results in treatment discontinuation [25,26,27,28].

In terms of correlation between MD changes and primary prevention efficacy only one study has directly linked tamoxifen-induced MD reduction to response and risk of BC developing [26]. In this study, the reduction in MD induced by tamoxifen is used to segregate women in function of the possible benefit they can get from the prophylactic treatment with this molecule. 46% of women receiving tamoxifen experienced a 10% or greater reduction in their MD after 12–18 months, which correlated with a 63% lower BC risk. On the contrary, those women receiving tamoxifen but showing less than a 10% reduction in their MD had no BC risk decrease. The study concluded that MD can be an excellent predictor of response to the preventive use of tamoxifen [26].

Finally, it is worth mentioning that hormone replacement therapy (HRT) used to ameliorate the symptoms of menopause increases breast cancer risk and mammographic density. HRT may consist of oestrogens alone or in combination with progestin. The possible role of MD in the relationship between HRT and BC has been studied [12] and a partial role of MD has been found since the association between HRT and BC risk is stronger in women with high MD [14].

## 4. Relevance of MD in the DCIS-to-ICD Transition

The most common non-invasive breast cancer lesion is ductal carcinoma in situ (DCIS), a highly heterogeneous pre-invasive lesion whose evolution is different in every patient. In some instances, DCIS can rapidly progress to the more aggressive form of invasive ductal carcinoma (IDC) if untreated or undertreated, whereas most of them will remain virtually unaltered for up to 20 years or will even not progress at all [29]. In this context, and considering that DCIS diagnosed patients are generally treated, there is a need to better define the particular risk of these patients to evolve to the invasive phenotype. Biologically, DCIS is defined by the proliferation of clonal cancerous epithelial cells that accumulate in the lumen of the ducts, but not migrating into the stroma of the mammary gland, thus preserving the myoepithelial cell layer and the basement membrane (BM) [30]. In fact, DCIS is usually regarded as a non-obligate previous step in the development of IDC, since the loss of myoepithelial cells and the breach of BM leads to an IDC, in which tumour epithelial cells invade the mammary stroma and eventually evolve to metastatic BC [30] (Figure 1B1). Epidemiologically, DCIS represents 20–25% of all new BC cases diagnosed [31] and this incidence is increasing as a result of an upgraded resolution of breast mammography [32]. Around 80% of the DCIS are identified by the presence of micro-calcifications and the remaining 20% by the detection of architectural deformation in mammography screening [33,34] (Figure 1B2).

Stromal cancer biology changes such as ECM remodelling, stromal cell alterations and chemical cues (hormones, cytokines and growth factors) correlate with patient outcome [35] and around 90% of these alterations occur during the DCIS phase [36]. In fact, these interactions between the epithelial and the stromal compartment clearly influence breast density and therefore its MD [6] already in the DCIS stage. While there is a lack of information about the association between MD and DCIS, in general terms it seems that high MD could correlate with the detection of DCIS lesions, although this association is less evident than with invasive breast cancer [37]. Thus, dense regions in mammographies have been tagged as susceptible areas of DCIS occurrence [38]. It has been reported that patients diagnosed with DCIS and presenting high MD (over 75%) have a higher probability to develop a second breast cancer, particularly in the contralateral breast when compared with low MD (under 25%) patients [39]. Other reports show a higher risk to develop DCIS in patients with MD over 75% [40]. To better classify DCIS lesions during diagnosis, a very recent communication correlates histopathological features of human patients with breast mammographies [41], evaluating mammographic digital images by principal component analysis taking into account stromal and glandular texture traits and MD. The authors thus provide the first preliminary results about the possibility to use mammographic patterns to improve the current DCIS classification. In addition, a very interesting recent study points towards a direct association between immune system activation and higher MD, since macrophages, dendritic cells, pro-inflammatory cytokines and B lymphocytes can increase MD in all cancer phases including pre-invasive DCIS lesions. B lymphocytes seem to be particularly important in the increase of MD in the benign lesions including DCIS [42].

## 5. Tumour Stroma as a Prognostic Factor

Classically, cancer research has focused mainly on the neoplastic cells within tumours. However, especially in the last years, it has become obvious that while tumour epithelial cells that have undergone genetic and epigenetic events are essential for the initiation of breast cancer, a variety of populations from the surrounding microenvironment also influence tumour progression [43]. The role of stromal factors in aiding cancer initiation, growth and progression has been well described [43,44,45,46,47], and during the last years it has also been suggested that the stroma components can have a crucial influence in the therapeutic outcome, and thus can be envisaged as possible relevant new targets [48].

It has been unanimously accepted that the stroma of a normal breast differs considerably from the one found in BC, but some trends in the normal tissue can be more predisposing to cancer development. Mammographically dense areas are associated with increased collagen I tissue deposition [49]. Using the Col1a1tmJae transgene model of reduced collagen proteolysis, it has been reported that a high collagen level in the stroma of murine mammary tissue results in a three-fold higher risk of developing BC with a more invasive phenotype [50]. Collagen density can exert this tumour-promoting role by at least two different mechanisms: i) By directly increasing the matrix stiffness and ii) by indirectly modulating mammary fibroblasts. In the first case, collagen would be diminishing the contractility of epithelial cells and thus altering focal adhesion and Rho signalling. In the second one, fibroblasts would start secreting aberrant soluble factors (among others transforming growth factor beta, epidermal growth factor and insulin-like growth factor) which would in turn modify the behaviour of epithelial cells [50].

Moreover, by next generating sequencing-based expression profiling, signatures from benign stromal proliferations have been identified that define stromal components of breast cancer with predictive value. Thus, genes known to be involved in hypoxic and angiogenic responses within tumours or in tumour-associated macrophage immune response have been identified in high MD tissue and related to a poor survival prognosis [51]. In addition, higher expression levels of cell adhesion and cell-cell contact genes have also been reported in non-tumoural stromal microenvironments in high MD tissues [52]. In a different study using samples from women undergoing prophylactic mastectomy because of their high BC risk profile, high MD tissues with no alterations in hormone receptor or Ki-67 marker status (and thus reputed as cancer-free) were described to have increased collagen deposition and changes in its organization, compared to low MD tissues [53]. These data highlight the importance of weighing both qualitative and quantitative stroma elements when evaluating the influence of mammographic density.

## 6. CAFs, MD, Cancer Progression and Chemoresistance

**Figure 3:**
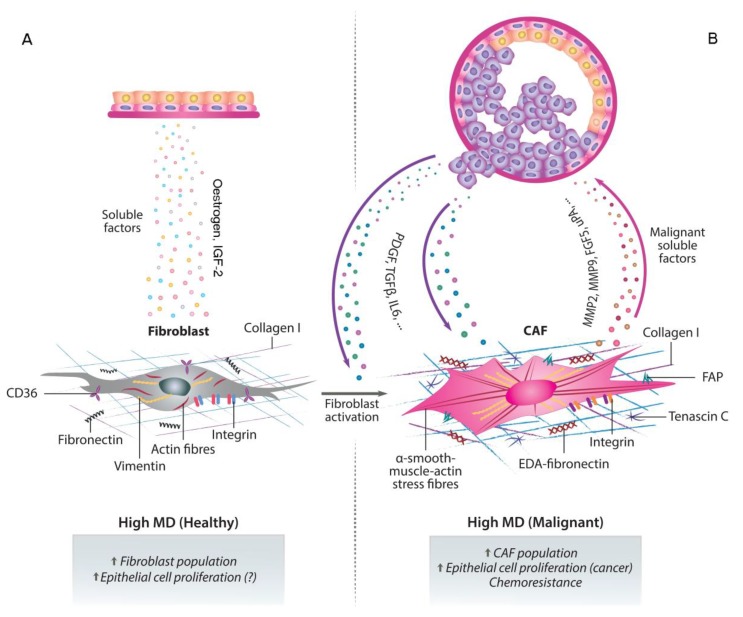
Paracrine crosstalk between fibroblasts and neighbouring epithelial cells. The abundance of stromal fibroblasts correlates with areas of high MD and stimulates epithelial cell proliferation by secreting copious soluble factors (**A**). The overstimulated epithelial cells, in turn, can undergo phenotypic changes and secrete fibroblast-activating factors. In these areas of high MD, the altered microenvironment can lead to the activation of fibroblasts into CAFs, which facilitate the growth and progression of the tumour cells. The secretion of growth factors, cytokines and proteases by CAFs establishes a positive feedback loop between both cell types which eventually leads to infiltration of immune cells and to chemoresistance. This pro-tumoural stroma also promotes angiogenesis, metastasis and therapy resistance (**B**).

Fibroblasts are the most abundant and active cell population of the breast stroma and so partially responsible for high MD. Besides, it is becoming increasingly clear that they are also prominent modifiers of cancer progression [48]. Activated fibroblasts are known as cancer-associated fibroblasts (CAFs) and are thought to be involved in tumour growth and metastasis [48]. CAFs can affect the phenotype of epithelial cells in a variety of ways, from cell-to-cell contacts to the secretion of aberrant soluble molecules of by altering critical ECM components [54]. It is well-known that alterations in stromal composition correlate with increased MD and it has been suggested that the numerous fibroblasts responsible for areas of high MD may be secreting soluble factors that would be inducing epithelial cell proliferation [55] (Figure 3A).

Fibroblasts can regulate different aspects of tumour biology and therefore play an important role in the different stages of breast tumour progression. Initially, in the early stages of tumour development, fibroblasts can inhibit proliferation by forming gap junctions among them [56] but in later phases, they can promote tumour growth and progression by secreting growth factors, cytokines and proteases, which leads to immune cell infiltration. This, in turn, will promote angiogenesis and metastasis [57]. Furthermore, CAFs can also secrete plasminogen activators and different MMPs, that can be used by the tumour in two different ways: to degrade ECM components and thus allow tumour expansion and promote angiogenesis; or to cleave soluble factors and cell adhesion molecules, which will result in an increased motility and EMT capacity [57] (Figure 3B).

Additionally, the existence of a crosstalk between stromal and epithelial cells can be deduced from the fact that highly dense stroma promotes epithelial cell proliferation in women showing high MD. A good candidate to participate in such a crosstalk is CD36, whose levels are significantly lower in fibroblasts from highly dense stroma than from low-density stromal fibroblasts from disease-free women [58]. This decrease in CD36 is particularly meaningful, since a similar downregulation of CD36 expression is observed in CAFs compared to fibroblasts from healthy reduction mammoplasty tissue, thus suggesting that it can be an early event in tumour formation [58].

The acquisition of resistance to therapeutic drugs by cancer cells seems to be also influenced by the tumour microenvironment. This progressive accumulation of mutations to generate the resistant population needs that cells that will develop the acquired genetic alterations to become resistant to a certain drug must obtain first a certain degree of protection from the lethal effects of the drug through a non-genetic mechanism. This protection of a pre-resistant cell population can be provided by the microenvironment, which would create the conditions needed by the cells to acquire all the genetic and epigenetic changes to become resistant [43,59].

In view of the central role CAFs play in the biology of BC, it is highly probable that they may also play a relevant role in the survival of tumour cells after the exposure to drugs. From co-culture and xenograft experiments, it has been shown that cell cycle arrest or senescence of stromal fibroblasts is critical for the sensitivity of the tumour to chemotherapy [60]. Fibroblasts from the stroma can also exert their influence on the chemosensitivity of tumour cells by indirect mechanisms, such as modulating ECM behaviour and stimulating integrin-mediated adhesion to fibronectin [61]. CAFs secrete type I collagen, which decreases chemotherapeutic drug uptake in tumours, favouring primary tumour cell proliferation and metastasis in multidrug-resistant murine breast cancer [62].

Increasing evidences have revealed that CAFs can cause endocrine, chemotherapy and targeted therapy resistance [63,64], including also anti-angiogenic therapy and TKI-targeted therapy [49,65]. Our own group has shown that HER2-positive CAFs secrete soluble factors, such as FGF5, that are able to switch the phenotype of HER2-positive breast cancer cells from sensitive to resistant to trastuzumab and lapatinib [66], in agreement with other results, obtained in 3D co-cultures of fibroblasts and breast cancer cells in which the later were protected from lapatinib [67]. CAFs have also been shown to induce trastuzumab resistance in HER2-positive BC cells by expanding the cancer stem cell population and activating several signalling pathways such as NFkB [68]. Furthermore, CAFs are also involved in resistance to tamoxifen [69]. Tamoxifen induces aromatase expression in CAFs, thus leading to the promotion of aggressive behaviour of breast tumours in response to tamoxifen, via the activation of a G protein coupled oestrogen receptor (GPER/GPR30) [70]. All these studies provide solid indications that preventing fibroblast activation can represent a novel therapeutic strategy in targeting tumour microenvironment.

## 7. Future Perspectives: CAFs as Therapeutic Targets and Improved Mammographic Monitoring

Traditionally, the development of new drugs against cancer has been centred on different trends of the tumour cell. Recently, the strategies for novel therapeutic and prevention strategies have focused rather on the tumour microenvironment than in the cancer cell itself. Among the various non-immune cells that surround a tumour, some stromal cells such as fibroblasts have been proven to play a critical role in promoting tumour proliferation, angiogenesis, invasion and metastasis [71].

Because the development of drug resistance seems to depend on the genetic stability of target cells, stromal cells, that are genetically more stable than tumour cells, have been proposed to be a more beneficial therapeutical target, since the possibility of acquiring drug resistance would be lower. At least 80% of stromal fibroblasts in breast cancer may have an activated phenotype [72], and since CAFs are genetically more homogeneous than cancer cells, they are less likely to acquire resistance to drugs, making them an attractive target for cancer therapy.

Several aspects of the biology of CAFs make these cells an attractive choice for effective anti-cancer therapies. Since PDGF and TGFβ play a key role in fibroblast activation, the development of inhibitors against these molecules has recently become a promising field of research [73]. Imatinib inhibits the tyrosine kinase activity of the PDGF receptor, so it has been used in the therapy of chronic myeloid leukaemia, among other cancers [73].Promising inhibitors of the TGFb signalling pathway at different levels have entered several clinical trials and shown encouraging results [74]. Yet another interesting target is the fibroblast activation protein (FAP). This membrane-bound protease is over-expressed in cells from the stroma and can enhance tumour growth in vivo [75]. This protein is the target of Sibrotuzumab, a humanized monoclonal antibody designed against it, but also of vaccines developed to generate an immune reaction to the FAP antigen [76]. An opposite strategy has been to exploit the serine protease activity of FAP to activate pro-toxins in the vicinity of the tumour [76].

Patients with an ER-negative BC showing the over-expression of a specific stromal gene signature show a worse response to neoadjuvant chemotherapy [75] thus supporting the concept that microenvironment can be an efficient target to improve their clinical response. [77]. The comparison between the transcriptome of normal fibroblasts and CAFs from different malignancies can lead to the identification of unique transcriptional signatures of potential clinical interest [78,79,80,81].

Despite the fact that clinical trials have been focused on the immune and vascular component of tumour microenvironment, new studies on targeting CAFs are appearing. The disruption of the communication pathway between stroma and tumour by inhibiting integrins is another strategy that has reached some clinical trials, although the drugs used have shown limited efficacy by now [82,83]. The first phase 1 and 2 clinical trials of Sibrotuzumab, an antibody targeting FAP, did not obtain a good outcome [84], and neither the inhibition of the protease activity of FAP with specific inhibitors has resulted in better survival rates for the patients [84]. Currently, there is one more study that is underway involving targeting of FAP. In the context of breast cancer, a phase 1 study evaluates the safety, pharmacokinetics and therapeutic activity of RO6874281 as monotherapy, RO6874281 combined with Trastuzumab, or RO6874281 combined with Cetuximab, for patients with breast and head and neck cancers (NCT02627274) [85]. In addition, several antifibrotic drugs (e.g., losartan, tranilast, pirfenidone), because of their capability to normalise the tumour microenvironment and to potentiate chemotherapy by enhancing the drug delivery, have been proposed as new promising drugs for dual use in clinical trials [54].

Finally, it has been postulated that changes in MD could be used as a biomarker for evaluating breast cancer prevention strategies [38] and as a surrogate biomarker for preventive and adjuvant endocrine therapies [22,25]. However, the use of MD in the clinical practice still requires the optimization of MD techniques and feasible in-depth computer-assisted analysis.

In this sense, recent MD analysis advances and digital image evaluation by principal component analysis, which provides more accurate information about mammographic traits, also propose MD patterns as prognosis and possibly follow-up tools in BC patients [41]. Other strategies include the use of ultrasound elastography (also known as strain imaging) to monitor the effects of chemotherapy on BC and peritumour stromal cells. This technique assesses the relative stiffness of a given tissue, as a reflection of the degree of necrosis, fibrosis and inflammation [86]. Thus, it may be an indicator of the response to chemo/hormonal therapy and helpful in monitoring the patient’s response to therapy, paying special attention to the stromal component (NCT01737970).

Therefore, the avenue of new and improved MD detection techniques, along with the joint therapeutic approach against the stromal and non-stromal cellular compartments of the tumour will provide clinicians with empowered tools to prevent BC and fine-tuning patient’s responses and follow-up.
